# White Rice Consumption and Risk of Colorectal Cancer Among Japanese Americans: The Multiethnic Cohort Study

**DOI:** 10.2188/jea.JE20200611

**Published:** 2023-04-05

**Authors:** Yuito Okada, Song-Yi Park, Lynne R. Wilkens, Gertraud Maskarinec, Yurii B. Shvetsov, Christopher Haiman, Loïc Le Marchand

**Affiliations:** 1 Population Sciences in the Pacific Program, University of Hawaii Cancer Center, Honolulu, Hawaii, USA; 2 Office of Public Health Studies, University of Hawaii, Honolulu, Hawaii, USA; 3 Department of Preventive Medicine, Keck School of Medicine, University of Southern California, Los Angeles, California, USA

**Keywords:** white rice, nutrition, colorectal cancer, Japanese, prospective study

## Abstract

**Background:**

White rice is a staple food for Japanese, a population at high risk for colorectal cancer (CRC). We investigated the association between white rice intake and CRC among Japanese Americans in the Multiethnic Cohort (MEC) study.

**Methods:**

The MEC study is a prospective study established in Hawaii and California in 1993–1996. Usual dietary intake was assessed using a validated quantitative food frequency questionnaire at baseline. Cox proportional hazards models were used to compute hazard ratios (HRs) and 95% confidence intervals (CIs) for quartiles of intake and to perform trend tests across sex-specific quartiles with adjustment for relevant confounders.

**Results:**

We identified 1,553 invasive CRC cases among 49,136 Japanese Americans (23,595 men and 25,541 women) during a mean follow-up of 19 years. White rice consumption was not associated with overall CRC incidence in men (*P_trend_* = 0.11) or women (*P_trend_* = 0.56). After excluding participants with a history of diabetes, the inverse associations were significant for CRC (*P_trend_* = 0.03, HR for quartile 4 [Q4] vs quartile 1 [Q1], 0.81; 95% CI, 0.64–1.03) and tumors of the distal colon (*P_trend_* = 0.006, HR for Q4 vs Q1, 0.66; 95% CI, 0.44–0.99) among men but not women.

**Conclusion:**

White rice consumption was not associated with an increased risk of overall CRC among Japanese Americans. An inverse association was observed with risk of CRC and distal colon cancer in men without a history of diabetes.

## INTRODUCTION

White rice is a primary staple food for many people of Japanese ancestry and a main component of the traditional Japanese diet. Rice (primarily refined rice, although brown rice has gained in popularity) is a major energy source for Japanese Americans.^1^ White rice contains less dietary fiber, vitamins, and minerals than brown rice,^2^ and it may increase the risk of chronic diseases.^3^ A meta-analysis has shown that white rice consumption may increase risk of type 2 diabetes (T2DM) in the Chinese and Japanese populations.^4^ In contrast, a report from a large population-based cohort study in Japan has suggested that rice intake was not associated with risk of cardiovascular mortality or morbidity.^5^

Colorectal cancer (CRC) is the third most commonly diagnosed cancer and the second leading cause of cancer death in men and women in the United States.^6^ Lifestyle factors, such as obesity,^7^^,^^8^ physical inactivity,^9^^,^^10^ smoking,^11^^,^^12^ alcohol consumption,^13^^,^^14^ and dietary patterns low in dietary fiber,^15^^,^^16^ calcium and vitamin D,^17^^,^^18^ and folate^19^^,^^20^ and rich in red meat^21^^,^^22^ may contribute to the rising risk for CRC across populations. In particular, Japanese Americans have experienced a very high CRC incidence and are at increased CRC risk compared to Caucasians as demonstrated in the Multiethnic Cohort (MEC) Study (age-adjusted RR 1.40; 95% confidence interval [CI], 1.21–1.61).^23^ However, the causes for this increased risk are not fully understood.

The evidence for an association of white rice consumption with CRC has been limited. For instance, no significant relationship between rice consumption and CRC was observed in a Japanese cohort study with 11 years of follow-up.^24^ In contrast, a case-control study in Fukuoka, Japan, revealed a reduced risk of CRC with rice intake.^25^ Previous studies on rice consumption and CRC have not distinguished white rice from other rice, such as brown rice. Therefore, the current analysis aimed to specifically investigate the association of white rice consumption with CRC risk among the Japanese American population of the MEC. We hypothesized that white rice consumption may significantly increase the risk of incident CRC in men and women because white rice increases risk for T2DM, which is a known risk factor for CRC.

## METHODS

### Study population

The MEC is a prospective cohort study established from 1993–1996 to examine lifestyle factors, in particular diet, related to cancer and other chronic diseases. In brief, a multiethnic population was recruited consisting of more than 215,000 adults aged 45–75 years residing in Hawaii and California. The participants completed a mailed baseline questionnaire on demographics and lifestyle, including diet. The study design has been described previously^26^ and was approved by the Institutional Review Boards of the University of Hawaii and the University of Southern California.

The current study consisted of the Japanese Americans enrolled in the MEC (*n* = 56,806). Participants who had CRC prior to cohort entry reported in the baseline questionnaire or by linkage to tumor registries (see below) (*n* = 823), implausible diets based on total energy intake (all individuals 3 or more robust SDs out of the range of the mean based on a truncated normal distribution) or its components (fat, protein, or carbohydrate intake ≥3.5 SDs out of the range of the mean) (*n* = 2,074), or missing information for continuous covariates (*n* = 4,773) were excluded from the current analysis. Missing information for categorical covariates was kept as a dummy variable. As a result, a total of 49,136 participants (23,595 men, 25,541 women) were analyzed in the present report.

### Dietary assessment

Dietary intake was assessed at baseline using a self-administered quantitative food frequency questionnaire (QFFQ) with more than 180 food items (https://www.uhcancercenter.org/for-researchers/mec-questionnaires), which was developed based on 3-day food records and validated against 24-hour recalls collected from random samples of each ethnic group.^27^ In the validation study, the average correlation coefficients between nutrient densities from the QFFQ and the 24-hour recalls among Japanese were 0.66 for men and 0.61 for women.

White rice consumption was derived from the reported frequency of intake and serving size for white rice items. Rice in mixed dishes, such as soups and rice flour, were not included. Frequencies of intake were assessed as eight choices (never/hardly ever, once a month, 2–3 times a month, once a week, 2–3 times a week, 4–6 times a week, once a day, or 2 or more times a day). The serving size was measured in three portion sizes, which were small (1/2 cup or 1 scoop or less), medium (1 rice bowl or 1 cup or 1 musubi), and large (2 rice bowls or 2 musubi or more). Daily white rice intake (g/day) was transformed into energy density (g/1,000 kcal/day). Intake of other foods, such as brown rice and meat, was estimated using the same method. Micronutrients were computed by converting grams per day of each food to amounts of the dietary component by applying a study-specific food composition table and summing across items.^27^

### Case ascertainment

Incident cases of invasive adenocarcinoma of the colon and rectum were identified through linkage with the Surveillance, Epidemiology, and End Results Program (SEER) state tumor registries in Hawaii and California. Case ascertainment was complete through December 31, 2013. Cases were limited to invasive adenocarcinoma with other types of CRC cases censored at the date of diagnosis. According to the International Classification of Disease (ICD) codes, the anatomical subsites were categorized as follows: C18.0–C18.9, C19, and C20 for CRC, C18.0–C18.5 for proximal colon tumors, C18.6–C18.7 for distal colon tumors, and C19 and C20 for rectal cancer. Tumors overlapping subsites (C18.8–C18.9) were excluded in the subsite-specific analyses. Deaths and causes of death were identified through linkage to the death certificate files in Hawaii and California and the National Death Index. The end of follow-up was determined as the earliest of these events: a diagnosis of CRC, death, or closure date of December 31, 2013.

### Covariates

The models included covariates from the baseline questionnaire: indicator variables for history of diabetes (yes or no), family history of CRC (yes or no), history of polyps of the intestines (yes or no), education (high school or less, vocational school or some college, bachelor degree, or graduate degree), nonsteroidal anti-inflammatory drugs (NSAID) use (current use, previous use, or no), multivitamin use (yes or no), and hormone replacement therapy in women (women only; current use, previous use, or no); and continuous variables for age (years), body mass index (BMI; kg/m^2^), cigarette smoking (pack-years), alcohol consumption (g/day), moderate to vigorous physical activity (hours/day), and intakes of total energy (kcal/day), red meat (g/1,000 kcal/day), processed red meat (g/1,000 kcal/day), dietary fiber (g/1,000 kcal/day), calcium from food and supplement (mg/1,000 kcal/day), folate from food and supplement (mcg/1,000 kcal/day), and vitamin D from food and supplement (IU/1,000 kcal/day). Moderate to vigorous physical activity (hours/day) was derived from self-reported time spent in these activities per week and was found to provide reasonable agreement with estimates of energy expenditure from doubly labelled water.^28^ Intakes of magnesium (mg/1,000 kcal/day), vitamin B6 (mg/1,000 kcal/day), vitamin B12 (mcg/1,000 kcal/day), n-3 PUFA (g/1,000 kcal/day), and sodium (mg/1,000 kcal/day) were considered as additional covariates; however, the results were unchanged by their adjustment and are not shown.

### Statistical analysis

White rice consumption was measured as energy density (g/1,000 kcal/day) and divided into sex-specific quartiles. Characteristics of participants are shown according to quartiles of white rice intake. Cox proportional hazards models of CRC were performed to compute adjusted hazard ratios (HRs) and 95% CIs. Separate models for men and women were fit, and age was used as the time metric. In adjusted models, covariates were selected a priori based on previous studies.^8^^,^^10^^,^^24^^,^^25^ The proportional hazards assumption was evaluated using Schoenfeld residuals and log-log survival plots. The lowest quartile of white rice consumption was used as the reference group. Trend tests were performed to examine dose-response relationships, where the trend variable took the value 1, 2, 3, or 4 for each quartile. A separate model was run using cases with cancer at each anatomical location (distal colon, proximal colon, or rectum), with the cases at other subsites considered as censored at time of diagnosis. The analysis was repeated with participants limited to those without a history of T2DM at baseline since participants with T2DM may have modified their diet as the results of their T2DM diagnosis; in particular, they may have been asked by their physicians to avoid eating white rice.^29^ All statistical tests were two-sided, and the significance level was 0.05. SAS software, version 9.4 was used (SAS Institute, Cary, NC, USA).

## RESULTS

We identified 1,553 incident cases of CRC after a mean of 19 years of follow-up: 867 cases among 23,595 men and 666 cases among 25,541 women (Table 1). The age-truncated (ages 45–95) incidence of CRC was 223 per 100,000 person-years in men and 189 per 100,000 person-years in women.

**Table 1.** Baseline characteristics for Japanese men and women according to white rice consumption

	White rice				*P value* ^a^
	Quartile 1	Quartile 2	Quartile 3	Quartile 4	
**Men (*n* = 23,595)**					
Number at risk, *n*	5,899	5,899	5,899	5,898	
White rice intake, range, g/1,000 kcal/day	0–84.2	84.2–137.1	137.1–204.0	204.0–571.4	
White rice intake, median, g/1,000 kcal/day	50.9	109.5	166.9	261.2	
Age at cohort entry, years, mean (SD)	61.4 (9.3)	61.2 (9.2)	60.4 (9.1)	59.9 (8.9)	<0.001
Body mass index, kg/m^2^, mean (SD)	25.1 (3.3)	25.3 (3.3)	25.3 (3.3)	25.3 (3.4)	<0.001
History of diabetes, *n* (%)	829 (14%)	677 (11%)	640 (11%)	657 (11%)	<0.001
Family history of colorectal cancer, *n* (%)	593 (10%)	573 (9.7%)	587 (9.9%)	546 (9.3%)	0.026
History of polyps of intestines, *n* (%)	603 (10%)	596 (10%)	605 (10%)	562 (9.5%)	0.52
Education, *n* (%)					
High school or less	1,940 (33%)	2,047 (35%)	2,177 (37%)	2,356 (40%)	<0.001
Vocational school/some college	1,766 (30%)	1,754 (30%)	1,791 (30%)	1,858 (31%)	
Bachelor’s degree	1,316 (22%)	1,353 (23%)	1,294 (22%)	1,142 (19%)	
Graduate degree	877 (15%)	745 (13%)	637 (11%)	542 (9.2%)	
Cigarette smoking, pack-years, mean (SD)	14.5 (16.8)	15.3 (16.8)	15.9 (16.9)	16.9 (17.3)	<0.001
Alcohol consumption, g/day, mean (SD)	14.8 (34.4)	11.7 (25.2)	10.7 (20.9)	8.3 (16.6)	<0.001
Moderate to vigorous physical activity, hr/day, mean (SD)	1.35 (1.36)	1.32 (1.34)	1.36 (1.39)	1.30 (1.37)	<0.001
NSAID use, *n* (%)					
Currently use	1,339 (23%)	1,301 (22%)	1,208 (20%)	1,147 (19%)	<0.001
Previously use	702 (12%)	670 (11%)	643 (11%)	615 (10%)	
No	3,858 (65%)	3,928 (67%)	4,048 (69%)	4,136 (70%)	
Multivitamin use, *n* (%)	3,087 (52%)	2,923 (49%)	2,758 (47%)	2,512 (43%)	<0.001
Food and nutrient intake					
Total energy, kcal/day, mean (SD)	2,355 (895)	2,313 (846)	2,290 (863)	2,215 (743)	<0.001
Energy from carbohydrate, kcal/day, mean (SD)	1,214 (493)	1,240 (476)	1,274 (491)	1,348 (452)	<0.001
Energy from protein, kcal/day, mean (SD)	350 (142)	337 (134)	323 (131)	292 (111)	<0.001
Energy from fat, kcal/day, mean (SD)	743 (340)	698 (304)	649 (290)	530 (233)	<0.001
Brown rice, g/1,000 kcal/day, mean (SD)	23.9 (52.1)	11.1 (29.6)	10.5 (33.0)	7.0 (28.5)	<0.001
Red meat, g/1,000 kcal/day, mean (SD)	19.5 (12.3)	19.7 (10.8)	18.9 (10.6)	16.1 (9.3)	<0.001
Processed red meat, g/1,000 kcal/day, mean (SD)	8.7 (6.6)	9.2 (6.4)	9.0 (6.1)	8.1 (5.8)	<0.001
Dietary fiber, g/1,000 kcal/day, mean (SD)	11.2 (4.1)	10.0 (3.3)	9.0 (3.1)	7.8 (2.8)	<0.001
Sodium, mg/1,000 kcal/day, mean (SD)	1,705 (420)	1,719 (370)	1,694 (369)	1,625 (375)	<0.001
Calcium^b^, mg/1,000 kcal/day, mean (SD)	452 (241)	408 (211)	363 (202)	306 (202)	<0.001
Folate^c^, mcg/1,000 kcal/day, mean (SD)	265 (169)	240 (154)	216 (148)	185 (140)	<0.001
Vitamin D^d^, IU/1,000 kcal/day, mean (SD)	180 (192)	166 (177)	152 (174)	134 (168)	<0.001

**Women (*n* = 25,541)**					
Number at risk, *n*	6,385	6,386	6,384	6,386	
White rice intake, range, g/1,000 kcal/day	0–66.8	66.8–110.1	110.1–165.9	165.9–609.4	
White rice intake, median, g/1,000 kcal/day	36.2	89.3	133.5	219	
Age at cohort entry, years, mean (SD)	60.2 (9.2)	60.6 (8.9)	60.4 (8.8)	60.3 (8.7)	0.15
Body mass index, kg/m^2^, mean (SD)	23.5 (3.9)	23.7 (3.8)	23.7 (3.8)	23.7 (4.1)	<0.001
History of diabetes, *n* (%)	594 (9.3%)	540 (8.5%)	515 (8.1%)	620 (9.7%)	0.004
Family history of colorectal cancer, *n* (%)	763 (12%)	717 (11%)	707 (11%)	700 (11%)	0.20
History of polyps of intestines, *n* (%)	374 (5.9%)	368 (5.8%)	382 (6.0%)	382 (6.0%)	0.94
Education, *n* (%)					
High school or less	2,158 (34%)	2,443 (38%)	2,606 (41%)	3,014 (47%)	<0.001
Vocational school/some college	1,960 (31%)	1,952 (31%)	1,909 (30%)	1,795 (28%)	
Bachelor degree	1,157 (18%)	1,063 (17%)	1,005 (16%)	856 (13%)	
Graduate degree	1,110 (17%)	928 (14%)	864 (13%)	721 (11%)	
Cigarette smoking, pack-years, mean (SD)	4.0 (9.3)	4.1 (9.2)	4.2 (9.4)	4.8 (10.3)	0.005
Alcohol consumption, g/day, mean (SD)	1.9 (7.7)	1.5 (6.1)	1.3 (5.8)	1.1 (4.9)	<0.001
Moderate to vigorous physical activity, hr/day, mean (SD)	1.12 (1.14)	1.08 (1.11)	1.05 (1.09)	1.02 (1.12)	<0.001
NSAID use, *n* (%)					
Currently use	854 (13%)	872 (14%)	774 (12%)	787 (12%)	<0.001
Previously use	702 (11%)	661 (10%)	645 (10%)	624 (9.8%)	
No	4,829 (76%)	4,853 (76%)	4,965 (78%)	4,975 (78%)	
Multivitamin use, *n* (%)	3,670 (57%)	3,503 (55%)	3,334 (52%)	3,171 (50%)	<0.001
Hormone replacement therapy, *n* (%)					
Currently use	2,215 (35%)	2,262 (35%)	2,301 (36%)	2,176 (34%)	0.19
Previously use	909 (14%)	910 (14%)	894 (14%)	874 (14%)	
No	3,261 (51%)	3,214 (50%)	3,189 (50%)	3,336 (52%)	
Food and nutrient intake					
Total energy intake, kcal/day, mean (SD)	1,918 (773)	1,933 (630)	1,742 (626)	1,734 (654)	<0.001
Energy from carbohydrate, kcal/day, mean (SD)	1,089 (466)	1,102 (388)	1,016 (383)	1,078 (422)	<0.001
Energy from protein, kcal/day, mean (SD)	290 (124)	287 (101)	253 (99)	238 (96)	<0.001
Energy from fat, kcal/day, mean (SD)	586 (285)	581 (233)	500 (215)	432 (197)	<0.001
Brown rice, g/1,000 kcal/day, mean (SD)	30.0 (53.4)	12.3 (27.9)	11.5 (31.9)	8.4 (30.8)	<0.001
Red meat, g/1,000 kcal/day, mean (SD)	15.3 (10.5)	17.2 (9.9)	17.0 (10.3)	15.2 (9.3)	<0.001
Processed red meat, g/1,000 kcal/day, mean (SD)	6.1 (5.2)	7.1 (5.3)	7.2 (5.3)	6.7 (5.5)	<0.001
Dietary fiber, g/1,000 kcal/day, mean (SD)	13.7 (4.3)	12.0 (3.5)	11.2 (3.3)	9.7 (3.1)	<0.001
Sodium, mg/1,000 kcal/day, mean (SD)	1,706 (441)	1,735 (389)	1,752 (416)	1,754 (458)	<0.001
Calcium^b^, mg/1,000 kcal/day, mean (SD)	708 (484)	623 (484)	623 (431)	540 (455)	<0.001
Folate^c^, mcg/1,000 kcal/day, mean (SD)	323 (204)	280 (173)	271 (176)	241 (182)	<0.001
Vitamin D^d^, IU/1,000 kcal/day, mean (SD)	227 (239)	194 (202)	194 (210)	180 (223)	<0.001

NSAID, nonsteroidal anti-inflammatory drug; SD, standard deviation.

^a^Determined by one-way analysis of variance (ANOVA) for continuous variables and Chi-square test for categorical variables.

^b^Calcium from food and supplement.

^c^Folate from food and supplement.

^d^Vitamin D from food and supplement.

Men had higher white rice consumption than women with quartile-specific medians (g/1,000 kcal/day) of 50.9, 109.5, 166.9, and 261.2 in men, respectively. The corresponding values in women were 36.2, 89.3, 133.5, and 219.0 g/1,000 kcal/day. The Japanese men with a high intake of white rice were more likely to be younger at cohort entry and less educated, to report more pack-years of cigarette smoking, a lower alcohol consumption, and to be less likely to use NSAID and multivitamins. As to diet, men in higher quartiles of white rice intake tended to have a lower intake of total energy, energy from protein and fat, brown rice, red meat, dietary fiber, sodium, calcium, folate, and vitamin D. Similarly, women with higher white rice consumption had less education, a greater pack-years of cigarette smoking, lower alcohol consumption, and less NSAID and multivitamin use. Hormone replacement therapy was not significantly different by group. Also, lower intakes of total energy, energy from protein and fat, brown rice, dietary fiber, calcium, folate, and vitamin D were observed in women who consumed more white rice. The prevalence of T2DM at baseline among the participants was 12% in men and 8.9% in women. Among men, those in the lowest quartile of rice intake had the highest prevalence of T2DM (14%), while in women, those in the highest quartile had the highest prevalence (9.7%).

Table 2 shows the results of the Cox regression analysis for white rice among men and women with the lowest quartile of intake as the reference group. None of the adjusted HRs for overall CRC were significant in men: quartile 2 (HR vs quartile 1: 0.94; 95% CI, 0.78–1.13), quartile 3 (HR vs quartile 1: 0.82; 95% CI, 0.67–1.00), quartile 4 (HR vs quartile 1: 0.87; 95% CI, 0.70–1.08), and in women: quartile 2 (HR vs quartile 1: 0.96; 95% CI, 0.77–1.20), quartile 3 (HR vs quartile 1: 1.04; 95% CI, 0.83–1.31), and quartile 4 (HR vs quartile 1: 1.04; 95% CI, 0.82–1.33). No significant trends for the total population were observed in men (*P_trend_* = 0.11) and women (*P_trend_* = 0.56). In terms of specific anatomical subsites of CRC in men, an inverse association in quartile 3 for distal colon cancer was significant (quartile 3 vs quartile 1: HR 0.61; 95% CI, 0.42–0.88) but no evidence for trend (*P_trend_* = 0.057). Similarly, no significant trends were observed in men for proximal colon cancer (*P_trend_* = 0.28), and rectal cancer (*P_trend_* = 0.74), and in women for proximal colon cancer (*P_trend_* = 0.54), distal colon cancer (*P_trend_* = 0.87) and rectal cancer (*P_trend_* = 0.44).

**Table 2.** Colorectal cancer hazard ratios (and 95% confidence intervals) according to quartiles for white rice consumption

	Men					Women				

	Quartile 1	Quartile 2	Quartile 3	Quartile 4	*P* value for trend	Quartile 1	Quartile 2	Quartile 3	Quartile 4	*P* value for trend
**Number at risk**	5,899	5,899	5,899	5,898		6,385	6,386	6,384	6,386	
**Colorectal Cancer**										
Number of cases/person-years	228/95,021	222/95,993	2,200/98,661	217/98,266		159/113,342	158/113,554	173/113,708	176/113,927	
Age Adjusted^a^	1.0 (reference)	1.01 (0.85–1.21)	0.91 (0.76–1.09)	1.01 (0.84–1.21)	0.76	1.0 (reference)	0.99 (0.79–1.23)	1.11 (0.90–1.37)	1.14 (0.92–1.41)	0.13
Multivariate Adjusted^b^	1.0 (reference)	0.94 (0.78–1.13)	0.82 (0.67–1.00)	0.87 (0.70–1.08)	0.11	1.0 (reference)	0.96 (0.77–1.20)	1.04 (0.83–1.31)	1.04 (0.82–1.33)	0.56
**Proximal colon**										
Number of cases/person-years	83/95,021	75/95,993	87/98,661	67/98,266		85/113,342	71/113,554	70/113,708	94/113,927	
Age Adjusted^a^	1.0 (reference)	0.95 (0.70–1.29)	1.09 (0.81–1.46)	0.87 (0.63–1.20)	0.61	1.0 (reference)	0.85 (0.62–1.16)	0.84 (0.62–1.15)	1.13 (0.85–1.52)	0.40
Multivariate Adjusted^b^	1.0 (reference)	0.88 (0.64–1.21)	1.00 (0.73–1.38)	0.76 (0.52–1.10)	0.28	1.0 (reference)	0.82 (0.60–1.14)	0.82 (0.60–1.13)	1.12 (0.80–1.56)	0.54
**Distal colon**										
Number of cases/person-years	79/95,021	79/95,993	53/98,661	72/98,266		43/113,342	45/113,554	64/113,708	53/113,927	
Age Adjusted^a^	1.0 (reference)	0.99 (0.72–1.35)	0.66 (0.47–0.94)	0.93 (0.68–1.28)	0.26	1.0 (reference)	1.01 (0.67–1.53)	1.44 (0.98–2.12)	1.20 (0.81–1.79)	0.15
Multivariate Adjusted^b^	1.0 (reference)	0.95 (0.69–1.31)	0.61 (0.42–0.88)	0.79 (0.54–1.15)	0.057	1.0 (reference)	0.94 (0.62–1.44)	1.27 (0.85–1.90)	0.93 (0.60–1.46)	0.87
**Rectum**										
Number of cases/person-years	64/95,021	65/95,993	59/98,661	74/98,266		26/113,342	41/113,554	37/113,708	28/113,927	
Age Adjusted^a^	1.0 (reference)	1.10 (0.80–1.52)	0.98 (0.71–1.36)	1.22 (0.90–1.67)	0.32	1.0 (reference)	1.61 (0.99–2.60)	1.62 (1.00–2.61)	1.29 (0.78–2.14)	0.38
Multivariate Adjusted^b^	1.0 (reference)	0.99 (0.69–1.40)	0.88 (0.61–1.28)	1.12 (0.75–1.66)	0.74	1.0 (reference)	1.67 (1.01–2.76)	1.60 (0.94–2.70)	1.30 (0.72–2.34)	0.44

^a^Adjusted for age at cohort entry (years).

^b^Adjusted for age at cohort entry (years), body mass index (kg/m^2^), history of diabetes (yes, no), family history of colorectal cancer (yes, no), history of polyps (yes, no), education (high school or less, vocational school/some college, bachelor degree, graduate degree), cigarette smoking (pack-years), alcohol consumption (g/day), moderate to vigorous physical activity (hr/day), nonsteroidal anti-inflammatory drug (currently use, previous use, no), multivitamin use (yes, no), hormone replacement therapy (women only: currently use, previous use, no), total energy intake (kcal/day), red meat (g/1,000 kcal/day), processed red meat (g/1,000 kcal/day), dietary fiber (g/1,000 kcal/day), calcium from food and supplement (mg/1,000 kcal/day), folate from food and supplement (mcg/1,000 kcal/day), vitamin D from food and supplement (IU/1,000 kcal/day).

As shown in Table 3, an analysis was performed among the participants without a history of T2DM (*n* = 44,064, 90% of the total population; 20,792 men and 23,272 women). The age-truncated incidence of CRC was 217 per 100,000 person-years in men and 143 per 100,000 person-years in women. A trend for CRC in men was significant (*P_trend_* = 0.028, quartile 4 vs quartile 1: HR 0.81; 95% CI, 0.64–1.03) but not in women (*P_trend_* = 0.69, quartile 4 vs quartile 1: HR 1.04; 95% CI, 0.81–1.34). In subsite analysis, a significant reduction in risk was observed for distal colon carcinomas in men: quartile 2 (HR vs quartile 1: 0.95; 95% CI, 0.68–1.33), quartile 3 (HR vs quartile 1: 0.54; 95% CI, 0.37–0.81), and quartile 4 (HR vs quartile 1: 0.66; 95% CI, 0.44–0.99) with a significant trend (*P_trend_* = 0.006). No significant HRs or trends were observed in women.

**Table 3.** Colorectal cancer hazard ratios (and 95% confidence intervals) by white rice intake quartiles in participants without a history of diabetes

	Men					Women				

	Quartile 1	Quartile 2	Quartile 3	Quartile 4	*P* value for trend	Quartile 1	Quartile 2	Quartile 3	Quartile 4	*P* value for trend
**Number at risk**	5,198	5,198	5,198	5,198		5,818	5,818	5,818	5,818	
White rice, range, g/1,000 kcal/day	0–85.4	85.4–138.2	138.2–204.7	204.7–571.4		0–67.0	67.0–110.1	110.1–165.0	165.0–609.4	
White rice, median, g/1,000 kcal/day	52.7	110.5	168	261.7		36.3	89.5	133.1	218.0	
**Colorectal Cancer**										
Number of cases/person-years	203/85,571	201/86,227	169/88,210	183/87,903		146/104,203	144/104,328	150/104,565	158/104,826	
Age Adjusted^a^	1.0 (reference)	1.00 (0.83–1.20)	0.86 (0.71–1.05)	0.94 (0.78–1.14)	0.30	1.0 (reference)	0.97 (0.77–1.22)	1.03 (0.83–1.29)	1.11 (0.89–1.38)	0.29
Multivariate Adjusted^b^	1.0 (reference)	0.95 (0.78–1.16)	0.78 (0.63–0.96)	0.81 (0.64–1.03)	0.028	1.0 (reference)	0.96 (0.76–1.21)	0.99 (0.78–1.26)	1.04 (0.81–1.34)	0.69
**Proximal colon**										
Number of cases/person-years	70/85,571	63/86,227	73/88,210	61/87,903		79/104,203	64/104,328	58/104,565	84/104,826	
Age Adjusted^a^	1.0 (reference)	0.92 (0.66–1.28)	1.05 (0.76–1.46)	0.91 (0.65–1.27)	0.79	1.0 (reference)	0.82 (0.59–1.13)	0.73 (0.52–1.02)	1.09 (0.80–1.47)	0.71
Multivariate Adjusted^b^	1.0 (reference)	0.88 (0.62–1.25)	1.00 (0.71–1.42)	0.81 (0.55–1.21)	0.48	1.0 (reference)	0.81 (0.58–1.13)	0.74 (0.52–1.05)	1.11 (0.78–1.58)	0.69
**Distal colon**										
Number of cases/person-years	71/85,571	71/86,227	43/88,210	55/87,903		39/104,203	43/104,328	58/104,565	49/104,826	
Age Adjusted^a^	1.0 (reference)	1.00 (0.72–1.39)	0.60 (0.41–0.87)	0.81 (0.57–1.14)	0.044	1.0 (reference)	1.05 (0.69–1.62)	1.42 (0.95–2.12)	1.22 (0.80–1.85)	0.18
Multivariate Adjusted^b^	1.0 (reference)	0.95 (0.68–1.33)	0.54 (0.37–0.81)	0.66 (0.44–0.99)	0.0063	1.0 (reference)	0.99 (0.64–1.54)	1.27 (0.83–1.94)	0.95 (0.60–1.52)	0.87
**Rectum**										
Number of cases/person-years	60/85,571	64/86,227	52/88,210	63/87,903		23/104,203	36/104,328	32/104,565	24/104,826	
Age Adjusted^a^	1.0 (reference)	1.10 (0.79–1.52)	0.93 (0.66–1.31)	1.10 (0.79–1.53)	0.79	1.0 (reference)	1.59 (0.96–2.65)	1.59 (0.95–2.64)	1.25 (0.73–2.13)	0.49
Multivariate Adjusted^b^	1.0 (reference)	1.03 (0.72–1.48)	0.82 (0.56–1.22)	1.00 (0.66–1.51)	0.70	1.0 (reference)	1.69 (0.99–2.88)	1.58 (0.90–2.27)	1.30 (0.69–2.44)	0.48

^a^Adjusted for age at cohort entry (years).

^b^Adjusted for age at cohort entry (years), body mass index (kg/m^2^), family history of colorectal cancer (yes, no), history of polyps (yes, no), education (high school or less, vocational school/some college, bachelor degree, graduate degree), cigarette smoking (pack-years), alcohol consumption (g/day), moderate to vigorous physical activity (hr/day), nonsteroidal anti-inflammatory drug (currently use, previous use, no), multivitamin use (yes, no), hormone replacement therapy (women only: currently use, previous use, no), total energy intake (kcal/day), red meat (g/1,000 kcal/day), processed red meat (g/1,000 kcal/day), dietary fiber (g/1,000 kcal/day), calcium from food and supplement (mg/1,000 kcal/day), folate from food and supplement (mcg/1,000 kcal/day), vitamin D from food and supplement (IU/1,000 kcal/day).

## DISCUSSION

This study is the first population-based study investigating the association of white rice consumption with CRC among Japanese Americans. This analysis in a large cohort with a long follow-up revealed that white rice consumption was not associated with an increased risk for overall CRC in men and women. However, after excluding participants who had a history of T2DM at baseline, an inverse association was observed for white rice with risk of overall CRC and distal colon cancer among men only.

The overall null association of white rice consumption with risk for CRC in MEC appears to be consistent with previous studies in Japanese.^24^^,^^25^ A large cohort study in Japan reported no association between rice consumption, predominantly white rice, and CRC.^24^ In that study, the trend tests for overall CRC were not significant in men (*P_trend_* = 0.18, quartile 4 vs quartile 1: HR 0.77; 95% CI, 0.56–1.07) or women (*P_trend_* = 0.31, quartile 4 vs quartile 1: HR 1.10; 95% CI, 0.71–1.68). In contrast, a case-control study in Fukuoka^25^ observed a significant inverse association of rice consumption across quintiles with CRC (*P_trend_* = 0.03, quintile 5 vs quintile 1: OR 0.71; 95% CI, 0.50–1.02) among men and women who were analyzed in the same model. In a subsite analysis, the *P* for trend was 0.61 for proximal colon, 0.08 for distal colon, and 0.06 for rectum. The studies didn’t show the results stratified by diabetes. Overall, these results suggest that white rice is not associated with CRC risk in Japanese population.

In the MEC, the prevalence of T2DM was high (12% in men and 9.8% in women), reflecting the prevalence of this disease in the Japanese American population in the United States.^30^ After excluding participants with a history of T2DM, white rice showed a significant inverse trend for distal colon cancer in men. A study in Japan showed that a Western dietary pattern was associated with distal CRC risk only in men.^31^ There was also a strong positive association between the ratio of fat/carbohydrate consumption as an energy source and risk of distal colon cancer. The distal colon may be more susceptible to the carcinogenic effect of diet than the proximal colon and rectum. In our study, the participants without a history of T2DM in quartile 1 for rice had the highest median for a ratio of fat/carbohydrate consumption (0.62 in men and 0.54 in women), in contrast to those in quartile 4 (0.39 in men and 0.41 in women). This ratio was much lower in the Japanese study, with a range of 0.10–0.20. Taken together, the participants who consume less white rice were more likely to consume protein and fat, such as red meat and processed meat, which could increase CRC risk. Furthermore, a study investigating diet quality in relation to CRC incidence in the MEC study has shown that all four diet quality scores were inversely associated with cancer risk for the distal colon and rectum but not for the proximal colon, even in Japanese men.^32^ These results support the idea that tumors of the distal colon may be more strongly related to diet among Japanese American men without a history of T2DM.

The biological plausibility for an association between white rice and a reduced risk of distal colon cancer remains unclear. There are two possible explanations related to either an indirect or direct mechanism of protection against CRC. First, white rice intake could be a proxy for the traditional Japanese diet, as consumed in Japan before the introduction of western foods. Japanese immigrants who consume more white rice are more likely to consume a more traditional Japanese diet.^1^ Homemade Japanese foods consist of white rice, miso soup, grilled fish, and pickles, which may improve general health among the Japanese population.^33^ Japanese Americans have generally adopted a more American diet, but older individuals, as represented in the current study, still consume a significant amount of traditional foods.^34^ Inflammation is considered a hallmark of CRC risk, as evidenced by NSAIDs reducing CRC risk, including among Japanese American men, by lowering inflammation in the large bowels.^35^ White rice and fish could minimize inflammatory reactions by replacing Western foods, such as processed foods and red meat, which potentially increases inflammation in the distal colon.^36^^–^^38^ In our study, the participants consumed fewer percent of energy from white rice (19% in men and 16% in women) than in Japan (29%).^5^ Taken together, the Japanese Americans who consumed white rice may be more likely to consume homemade Japanese foods and fewer pro-inflammatory western foods.

Second, starch may directly ameliorate the gut microbiome in favor of a profile that may be protective against CRC.^39^ Starch is a main nutritional component of white rice and could have biological functions similar to dietary fiber after being converted into resistant starch. Although risk reduction from starch remains controversial,^40^ some studies report an inverse relation with CRC.^41^ In vivo, starch may improve the microbiome and total stool composition and stool volume to promote shifts in microbial diversity that may reduce inflammation by controlling COX-2, NF-kB, TNF-a, and IL-1b.^42^

The present study had several strengths, such as a population-based prospective design with a large number of participants and identification of cancer cases through high-quality tumor registries. Refined rice was distinguished from brown rice, and results were adjusted for a set of comprehensive covariates of CRC risk. The long follow-up allowed for the identification of a substantial number of cases. The limitations of the study include the exclusion of participants (13.5%) who had missing data, which may have introduced selection bias. White rice consumption was measured using a self-reported questionnaire, a possible source of information bias. However, misclassification due to measurement error is more likely to be non-differential, biasing the HRs toward the null.

In conclusion, this prospective analysis in the MEC suggests that, overall, white rice consumption is not associated with an increased incidence of CRC among Japanese Americans men and women. After excluding the participants with a history of T2DM at baseline, a significant inverse association was identified for overall CRC and distal colon cancer in men but not in women. This analysis may support the hypothesis that a diet with a large proportion of energy from white rice may be protective against CRC in men without a history of T2DM. However, it is not clear which component of a white rice-based diet, such as a high intake of resistant starch or a low intake of red meat, or other related lifestyle factors are responsible for this observation and why this benefit does not extend to women.
